# Estimation of transpulmonary driving pressure using a lower assist maneuver (LAM) during synchronized ventilation in patients with acute respiratory failure: a physiological study

**DOI:** 10.1186/s40635-024-00674-z

**Published:** 2024-10-04

**Authors:** Ling Liu, Hao He, Meihao Liang, Jennifer Beck, Christer Sinderby

**Affiliations:** 1 Jiangsu Provincial Key Laboratory of Critical Care Medicine, Department of Critical Care Medicine, School of Medicine, Zhongda Hospital, Southeast University, Nanjing, 210009 China; 2 Department of Critical Care, Keenan Research Centre for Biomedical Science of St. Michael’s Hospital, St. Michael’s Hospital, 30 Bond Street, Toronto, ON M5B1W8 Canada; 3 Department of Pediatrics, University of Toronto, Toronto, Canada; 4 Institute for Biomedical Engineering and Science Technology (iBEST), Ryerson University and St-Michael’s Hospital, Toronto, Canada; 5 Interdepartmental Division of Critical Care Medicine, Department of Medicine, University of Toronto, Toronto, Canada

**Keywords:** Diaphragm electrical activity, Neurally Adjusted Ventilatory Assist, Simulated Neural Pressure Support, Transpulmonary pressure, Lower assist maneuver

## Abstract

**Background:**

We previously showed in animals that transpulmonary driving pressure (PL) can be estimated during Neurally Adjusted Ventilatory Assist (NAVA) and Neural Pressure Support (NPS) using a single lower assist maneuver (LAM). The aim of this study was to test the LAM-based estimate of PL (PL_LAM) in patients with acute respiratory failure.

**Methods:**

This was a prospective, physiological, and interventional study in intubated patients with acute respiratory failure. During both NAVA and simulated NPS (high and low levels of assist), a LAM was performed every 3 min by manually reducing the assist to zero for one single breath (by default, ventilator still provides 2 cmH_2_O). Following NAVA and NPS_SIM_ periods, patients were sedated and passively ventilated in volume control and pressure control ventilation, to obtain PL during controlled mechanical ventilation (PL_CMV). PL using an esophageal balloon (PL_Pes) was also compared to PL_LAM and PL_CMV. We measured diaphragm electrical activity (Edi), ventilator pressure (PVent), esophageal pressure (Pes) and tidal volume. PL_LAM and PL_Pes were compared to themselves, and to PL_CMV for matching flows and volumes.

**Results:**

Ten patients were included in the study. For the group, PL_LAM was closely similar to PL_CMV, with a high correlation (*R*^2^ = 0.88). Bland–Altman analysis revealed a low Bias of 0.28 cmH_2_O, and 1.96SD of 5.26 cmH_2_O. PL_LAM vs PL_Pes were also tightly related (*R*^2^ = 0.77).

**Conclusion:**

This physiological study in patients confirms our previous pre-clinical data that PL_LAM is as good an estimate as PL_Pes to determine PL, in spontaneously breathing patients on assisted mechanical ventilation.

*Trial registration* The study was registered at *clinicaltrials.gov* (ID NCT05378802) on November 6, 2021

**Supplementary Information:**

The online version contains supplementary material available at 10.1186/s40635-024-00674-z.

## Background

To minimize lung injury during mechanical ventilation, there is currently much interest in knowing the transpulmonary driving pressure (PL) at the bedside [1]. By convention, PL is measured as the difference between airway pressure and pleural pressure, the latter usually estimated by esophageal pressure (Pes) (for both controlled mechanical ventilation and partial-ventilatory assist).

The PL measured during partial ventilatory assist (ventilator + patient) is the same as the PL measured during controlled mechanical ventilation (ventilator alone), when airway flow and volume are matched, according to the equation of motion [2–4]. Hence, Pes-based measurements of PL obtained during controlled mechanical ventilation (PL_CMV) or during assisted modes should be gold-standards for comparison of new PL-estimates, as long as inspiratory flows and volumes are similar.

We previously showed in animals that it is possible to estimate PL during Neurally Adjusted Ventilatory Assist (NAVA) and Neural Pressure Support (NPS) using a single lower assist maneuver (LAM) [2]. A LAM is a maneuver that is achieved by acutely lowering the assist for one single breath. The rationale is to obtain an “unassisted” breath, that can be compared to an assisted breath when full support is resumed [2]. The LAM breath is never truly “unassisted” because of the ventilator default, which provides 2 cmH_2_O, despite a setting of 0 cmH_2_O.

In a small animal model with both resistive and elastic loads, the LAM-based estimate of PL (PL_LAM) demonstrated a strong correlation to PL_CMV (*r* = 0.83), with a low bias and standard deviation (bias = 0.49 cmH_2_O and 1.96 * SD = 3.09 cmH_2_O) [2]. As described in detail in reference 2 and in “Methods”, PL_LAM is the ratio of the difference in ventilator pressure between the two assist levels, divided by the fraction of the patient’s contribution to tidal volume [2, 5, 6].

The aim of the present physiological study was to reproduce our findings from the pre-clinical model: we wanted to determine if PL_LAM is valid in intubated, spontaneously breathing patients, with two modes of neurally-synchronized mechanical ventilation: (i) NAVA and (ii) a simulated Neural Pressure Support, NPS_SIM_. We compared PL_LAM and PL_Pes (measured during spontaneous modes) to PL_CMV (measured during controlled ventilation), with matching flow and volume.

## Methods

The protocol was approved by Institutional Ethics Committee of Zhongda hospital (Approval Number: 2021ZDSYLL216-P01, September 1, 2021), and informed consent was obtained from the patients or next of kin. The trial “Predict Transpulmonary Pressure through ZAM”) was registered at clinicaltrials.gov (ID NCT05378802) on November 6, 2021. The procedures were followed in accordance with the ethical standards of the responsible committee on human experimentation (institutional or regional) and with the Helsinki Declaration of 1975.

### Patients

Patients were eligible for inclusion if they were (i) 18–85 years old, (ii) in respiratory failure on invasive mechanical ventilation; (iii) able to tolerate pressure support ventilation, (iv) under light sedation with RASS between − 2 and 1.

Patients were not eligible if (i) there was a contraindication for nasogastric tube insertion (history of esophageal varices, gastroesophageal surgery in the previous 12 months or gastroesophageal bleeding, international normalized ratio > 1.5 and activated partial thromboplastin time > 44 s), (ii) any disease affecting spontaneous breathing.

### Measurements

Patients were instrumented with a naso-gastric feeding-tube capable of measuring diaphragmatic electrical activity (Edi), esophageal (Pes) and gastric (Pga) pressures (Neurovent Research Inc, Toronto, Canada). The following measurements were recorded simultaneously: flow and ventilator pressure (PVent), Edi, Pes, and Pga. See Liu et al. [6] for more details on signal acquisition.

### Protocol

**Fig. 1:**
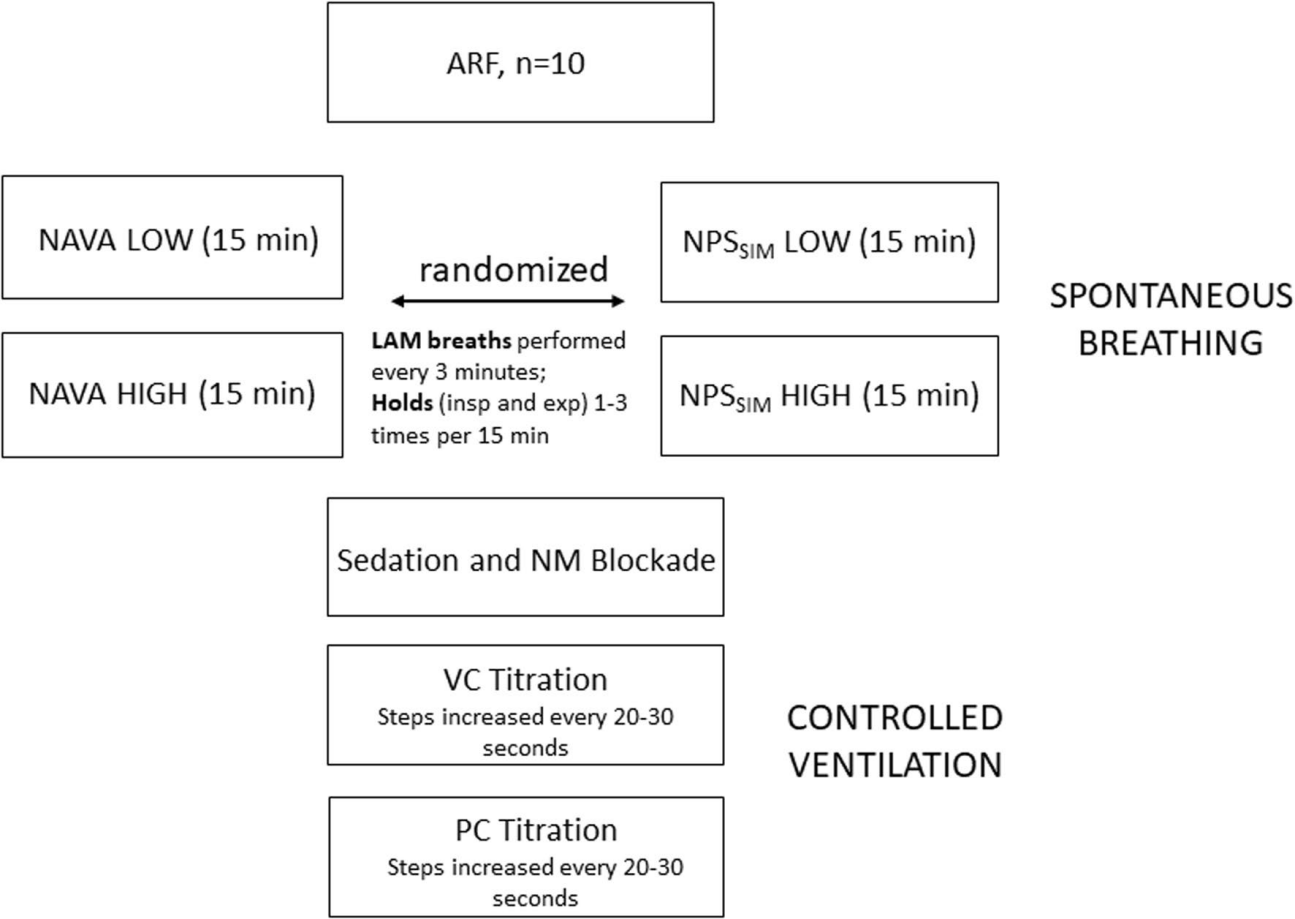
Schematic representation of experimental protocol. After inclusion, subjects (*n* = 10) were randomized to initially receive either two, 15-min periods of NAVA at two NAVA levels (NAVA LOW and NAVA HIGH) or two, 15-min periods of NPS_SIM_ at two pressures (NPS_SIM_ LOW and NPS_SIM_ HIGH) and then switched to the other mode. LAM breaths were performed manually (an acute lowering of the NAVA level to zero) every 3 min per 15-min ventilation period; inspiratory and end-expiratory holds were performed 1–3 times during the 15-min periods. Patients were then sedated/paralyzed and ventilated with stepwise increases in pressure control and volume control, with steps increased every 20–30 s. See “Methods” for more details

The protocol (Fig. 1) consisted of the following steps:

(i) Two NAVA periods (“Low” and “High” NAVA levels, 15 min each).
(ii) Two NPS_SIM_ periods (“Low” and “High” pressure support levels, 15 min each).
(iii) Heavy sedation/paralysis.
(iv) Step-wise increases in volume control (VC), and step-wise increases in pressure control (PC), every 20–30 s.

LAM breaths were performed during the NAVA and NPS_SIM_ runs, at least 5 per 15-min period.

Patients were ventilated with a Servo-i ventilator (Maquet, Solna, Sweden). During spontaneous breathing modes, patients were studied under light sedation with RASS between − 2 and 1. The purpose of using “high” and “low” levels of assist was to ensure a wide range of respiratory efforts, and to demonstrate the validity of the new PL-estimate (PL_LAM) over a broader range.

NAVA was used conventionally as previously described [7, 8], and NPS_SIM_ was provided using the NAVA mode at a maximum NAVA level (15 cmH_2_O/µV) with upper pressure-limit adjusted to target a PS level above PEEP [9–11]. The ventilator pressure levels studied are presented in the results (Fig. 4). There was no intention to compare the modes statistically, they were both included to test the PL_LAM estimate with different ventilator pressure profiles.

Lower Assist Maneuver (LAM): The LAM maneuver was performed by manually lowering the NAVA level to 0 cmH_2_O/µV, for one single breath [2]. Note that the Servo-i ventilator provides a minimal pressure of 2 cmH_2_O during the LAM, this is built-in to the machine and not adjustable.

Following the NAVA and NPS_SIM_ runs, spontaneous breathing was eliminated by deeply sedating (RASS − 3 to − 4) with propofol (2–3 mg/kg h) and paralyzing by intravenous injection bolus of vecuronium bromide (2–4 mg). Control mechanical ventilation at different levels allowed us to obtain breaths for matching the spontaneous breathing titrations (in terms of flow and volume). Absence of Edi (close to 0 µV) confirmed that the diaphragm was not active during CMV.

*Pressure control (PC) mode* and *volume control mode (VC)* were used to “match” breaths of similar volumes/flow as obtained during the NAVA or NPS_SIM_ runs (“matching” refers to later, off-line analysis, of assisted breaths and CMV breaths, see below “Analysis (off-line)”). In both modes, the assist was progressively increased in a step-wise fashion, every 20–30 s. On average for the group, 17 ± 4 steps in PC were performed between 5.5 ± 2.3 and 23.8 ± 6.5 cmH_2_O. In VC mode, on average 19 ± 6 steps were performed and ranged between 22.6 ± 6 and 50.7 ± 12.4 LPM.

### Analysis (off-line)

**Fig. 2:**
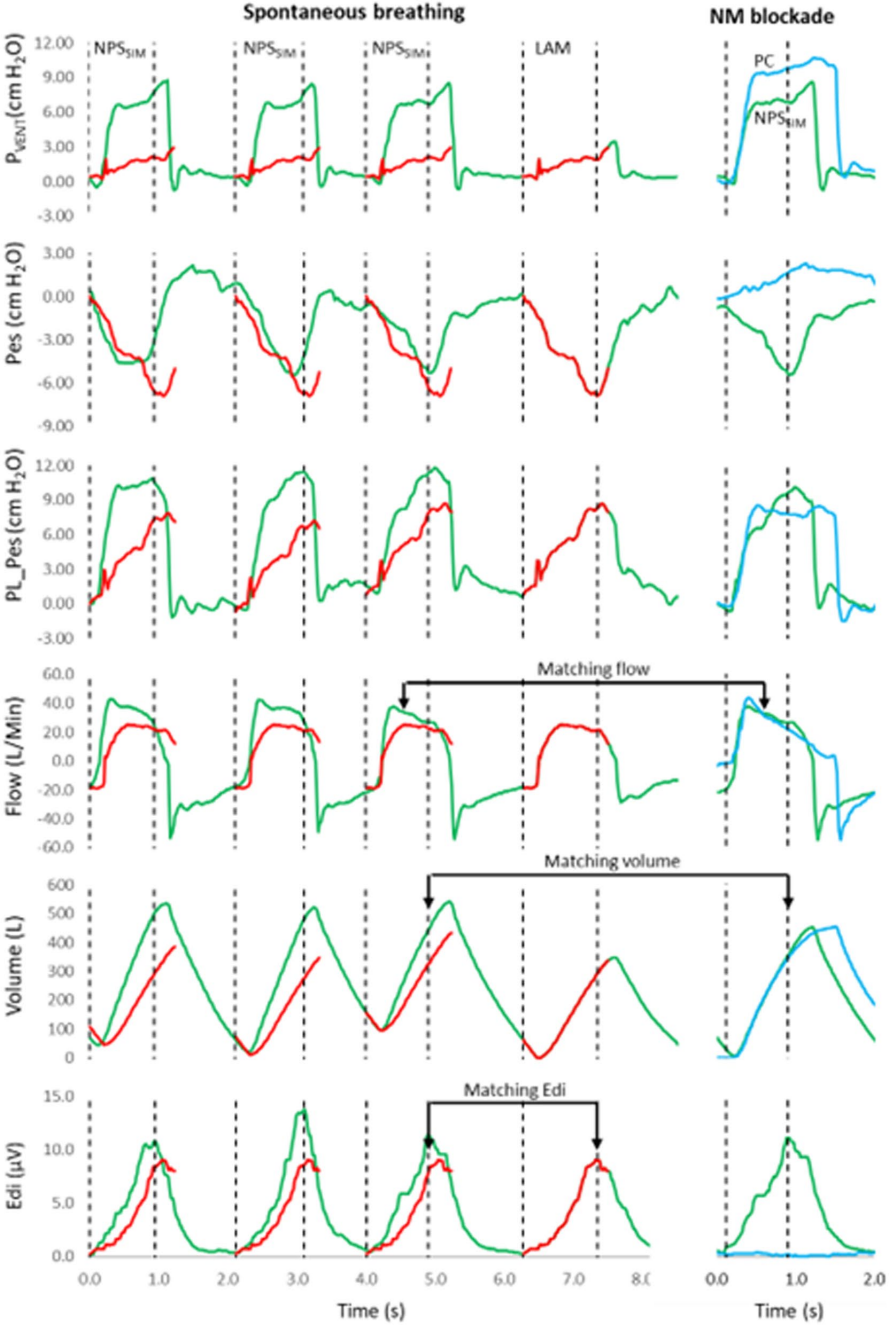
Examples of recorded and calculated waveforms to demonstrate experimental protocol, breath types, and breath matching (assisted, LAM, and controlled mechanical ventilation). Waveforms are obtained from one representative subject breathing with NPS_SIM_. From top to bottom, PVent, Pes, PL_Pes, flow, volume, Edi. Green waveforms indicate assisted breaths, red waveforms LAM breaths, and blue waveform the CMV breath. Vertical dashed black lines indicate the start and peak of Edi waveform. Examples of matching Edi waveforms for assisted and LAM breaths are indicated, as well as the matching flow and volume for assisted breaths and the CMV breath. All signals were continuously collected from the Servo-tracker. PL_Pes was mathematically produced by digital subtraction of the Pes waveform from the PVent waveform

**Fig. 3:**
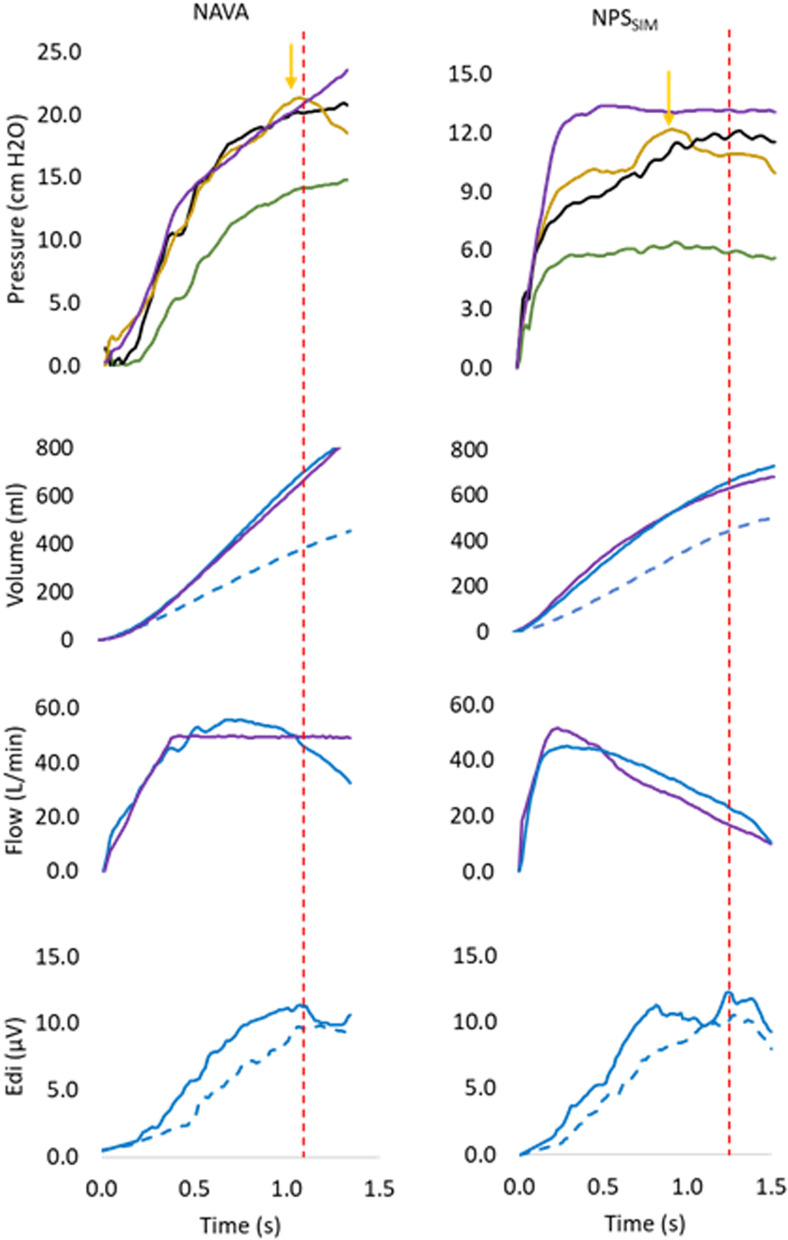
Examples of recorded waveforms to demonstrate off-line analysis. Waveforms are obtained from one representative subject breathing with NAVA (left panels) and NPS_SIM_ (right panels). Pressure panel: for both modes, the inspiratory portion of single breaths are displayed for PL_LAM (black solid); PL_Pes (yellow solid); PL_CMV (purple solid); PIVent (green solid), the latter calculated as the mathematical difference between PVent during assisted breath and PVent during the LAM. Volume panel: LAM breath (dashed blue line), assisted breath (blue solid line) and volume control breath (purple solid line) are plotted. PVBC was calculated as LAM volume/assisted volume (at peak Edi). For NAVA, assisted volume (blue solid) and matched volume control (purple solid) are superimposed to demonstrate matching of the breaths. For NPS_SIM_, volume during pressure control (purple solid) are superimposed on the assisted breath (solid blue). Flow panel: the corresponding flow waveforms are plotted for spontaneous modes (solid blue) and controlled modes (solid purple) and show examples of matching waveforms. Edi matching: Edi curves for LAM breaths (dashed blue) and assisted breaths (solid blue) were evaluated for their similarity (regression analysis with inclusion as defined in “Methods”). Vertical dashed red line indicates the time point of peak Edi, to where most variables are calculated, with the exception of PL_Pes, calculated to its peak (yellow arrow)

As previously described [2], the analysis was performed off-line, after the experimental data was collected. Analysis was performed both automatically and manually. Figure 2 demonstrates waveforms obtained in one subject for three types of breaths during NPS_SIM_, one LAM maneuver, and CMV. Figure 3 shows another example of single-breath waveforms with further explanations of the analysis.

#### Automated analysis

From the Edi waveform, two time-points (start and peak of Edi, Fig. 2, most bottom panel, vertical dashed lines) for the assisted breaths and the LAM breaths were detected automatically. Edi onset and Edi peak (also shown in Fig. 3, bottom panels, vertical dashed red line) was obtained by finding the “state” of the ventilator (a digital signal collected from the Servo-I ventilator). Hence, the onset of ventilator pressure and inspiratory flow, as well as their values at peak Edi, were also automatically detected and stored.

Initial screening criteria for including LAM breaths and assisted breaths in the analysis were: tidal volume > 100 ml, and peak Edi > 1 µV. Regression analysis of (inspiratory) Edi between the LAM and the assisted breath must have had *R*^2^ > 0.8 and must have included a minimum of at least 192 ms. The slope values needed to be 0.7–1.4 and the intercept less than ± 2 µV. The assisted breaths that had an “Edi-matched” LAM were then stored for later comparison to the breaths obtained during the PC or VC mode.

Calculation of PL_LAM: For Edi-matched breaths, for each LAM period, the LAM-based estimate of PL was calculated as previously described by Liu [2]:

$${\text{PL}}\_{\text{LAM}} = {\text{PIVent}}/\left( {1 - {\text{PVBC}}^{2} } \right),$$

where PIVent = PVent (assist) − PVent (LAM), and PVBC = Volume LAM/Volume Assist [5, 6].

PL_LAM was calculated to peak Edi. All breaths with PVBCs up to 0.85 were included. When PIVent (numerator of the equation) was 1–5 cmH_2_O, PVBC up to 0.90 was permitted.

Calculation of PL_Pes: During assisted modes (NPS_SIM_/NAVA), the PL_Pes waveform was created by digital subtraction of Pes from PVent, examples are provided in Figs. 2 and 3 [3]. The start of the PL_Pes waveform was adjusted to zero for each breath (i.e., driving pressure). PL_Pes was calculated to its peak (see Fig. 3, yellow arrow indicates peak PL_Pes). The influence of expiratory muscles’ relaxation on inspiratory Pes was minimized by an algorithm based on Pes baseline values just prior to inspiration for the LAM and the assisted breaths. If Pes baseline difference was > 2 cmH_2_O, the samples were discarded. For PL_Pes and PL_LAM calculations, any negative deflection in airway pressure (PVent) immediately prior to the onset of assist, was added to both.

#### Manual analysis

Manual analysis was performed to validate the automated analysis. Comparing the automated analysis to the manual analysis also allowed the minimization of selection bias.

The steps in the manual analysis were:

(i) Comparing LAM breaths and assisted breaths for matching Edi, by visual inspection of superimposed Edi curves for both breath types
(ii) Comparing CMV breaths to selected assisted breaths (from above) for matched flow/volume curves.

More specifically, flow and volume from the different step-wise increases in CMV were superimposed and compared to the flow and volume curves from each assisted breath, until the best match was obtained visually (see Fig. 2). A new average was calculated for PL_LAM and PL_Pes for the matched assisted breaths. As with PL_Pes, PL_CMV was calculated as Pvent-Pes (above PEEP). PL_CMV was measured up to the volume where the peak Edi occurred during the matched assisted breath (see Fig. 3, see vertical red dashed lines). At least 10 breaths visually matching flow and volume curves were required.

Respiratory system compliance and resistance were calculated from the data collected during VC at the end of the study protocol, see Electronic Supplementary Material, Methods section.

### Statistics

For comparing PL estimates to PL_CMV, and PL_LAM to PL_Pes, Bland–Altman plots were used, and bias is reported as well as the limits of agreement = 1.96 × standard deviation (1.96SD). Regression analysis was used, and we report determination coefficients (*R*^2^), slopes and intercepts. Statistical analysis was performed using Sigma Stat (v.10).

## Results

Intermittent end-expiratory holds revealed an excellent correlation between Pes and Paw (*R*^2^ = 0.91), and regression showed Paw = 1.06 × Pes − 1.74 (cmH_2_O).

Ten male patients with ARF (mean age 71 ± 13 yrs, height 171 ± 9 cm, and weight 71 ± 4 kg), of varying etiology and respiratory compromise were studied (Table 1).

**Table 1:** Patient characteristics

Patient number	Main diagnosis	pH	PaCO_2_ (mmHg)	PaO_2_ (mmHg)	FiO_2_	Compliance (ml/cmH_2_O)	Resistance (cmH_2_O/l/s)
1	Intracerebral hemorrhage	7.20	51.5	81.9	0.45	40.9	25.1
2	Pneumonia	7.49	32.3	89.3	0.4	47.4	6.7
3	Spinal cord injury	7.45	34.8	104.8	0.3	71.2	12.0
4	AECOPD	7.44	39.3	87	0.45	25.7	19.6
5	Polytruama	7.34	50.8	96.9	0.3	44.3	15.7
6	Urinary tract infection	7.42	33.8	78.2	0.25	74.3	16.9
7	Intestinal obstruction	7.40	35.1	100.7	0.35	33.3	10.9
8	Urinary tract infection	7.44	41.3	89.4	0.3	25.3	14.2
9	Intracerebral hemorrhage	7.37	41.9	87.1	0.3	35.6	16.4
10	Pneumonia	7.45	29.3	100.7	0.3	34.3	21.9
Mean		7.40	39.0	91.6	0.3	43.2	15.9
SD		0.08	7.5	8.8	0.1	17.1	5.4

In total, 200 LAM breaths were performed (5 per 15-min ventilation period, 2 modes × 2 levels of assist, in 10 patients). One hundred and seventy-six (176) LAMs were included in the analysis (9 LAMs did not meet the initial inclusion criteria (VT, Edi), and 15 LAMS did not find an assisted match. In total, 3212 Edi-matches (44%) passed inclusion criteria, out of 7250 comparisons between the LAM and the assisted breaths. The manual analysis included 1551 breaths (i.e., 1551 assisted breaths had a match to CMV breaths).

**Fig. 4:**
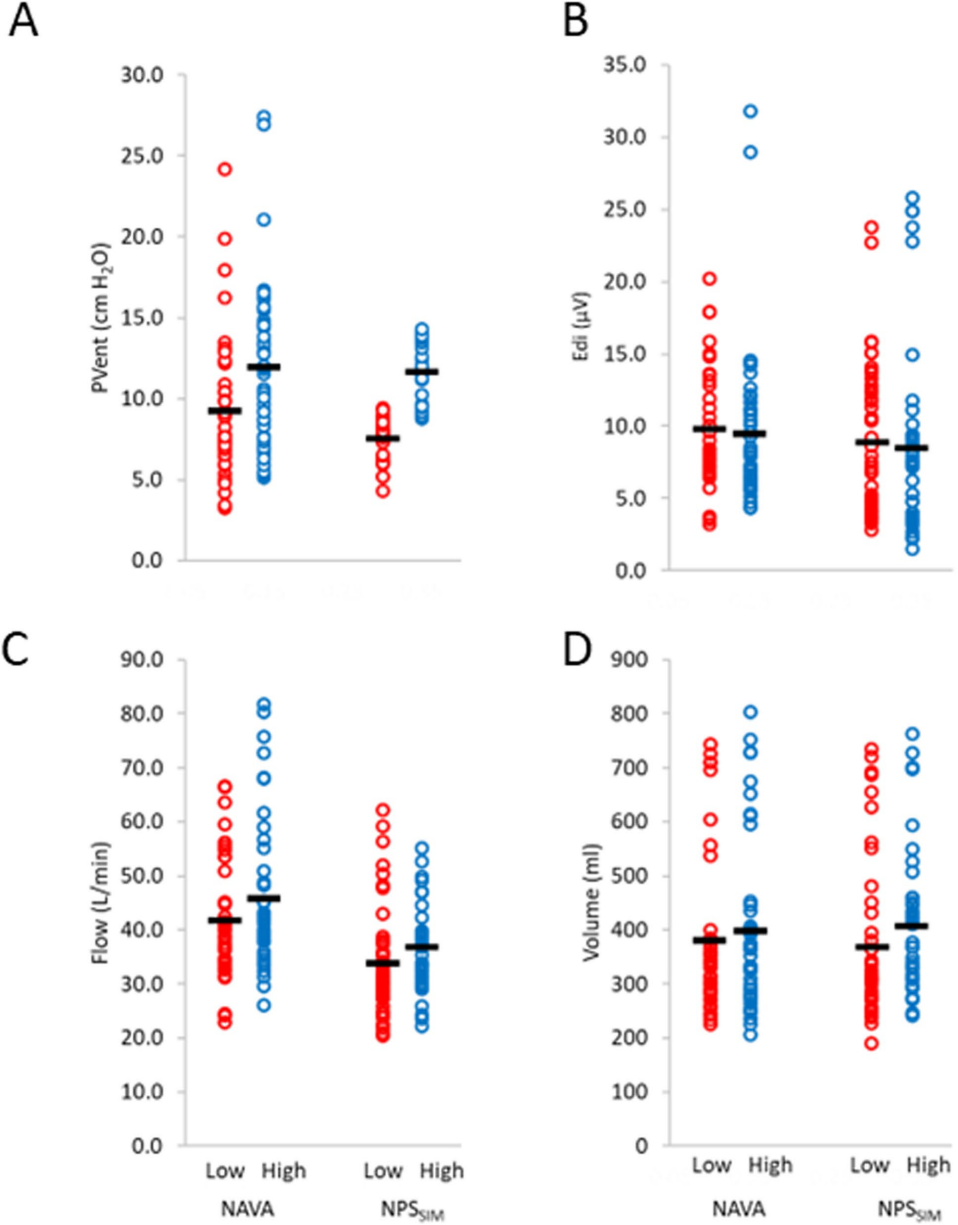
Group mean data for measured pressures, diaphragm electrical activity, flow, and volume during the four ventilator conditions (NAVA and NPS_SIM_). **A**–**D** Show individual patient’s data (*n* = 176 open symbols) and mean data (short horizontal black bar, *n* = 40) for the four ventilator conditions: NAVA low (red), NAVA high (blue symbols), NPS_SIM_ low (red), and NPS_SIM_ high (blue) for PVent (**A**), Edi (**B**), flow (**C**), and volume (**D**). All data are presented for the assisted breaths (no LAM data). PVent and Edi are calculated to peak Edi, volume is calculated for the full breath, and peak flow are reported

Figure 4 demonstrates the measured variables during the four ventilator periods (spontaneous modes), for individual patient’s data (*n* = 176, red symbols for low levels of assist, blue symbols for higher levels), as well as the mean for the group (short black horizontal bar). For the group, assist levels (PVent) were higher during the “High” periods, as well as a reduced Edi peak, increased flow, and increased volume. The figure also demonstrates the wide range in assist levels and respiratory demand for this patient group.

**Fig. 5:**
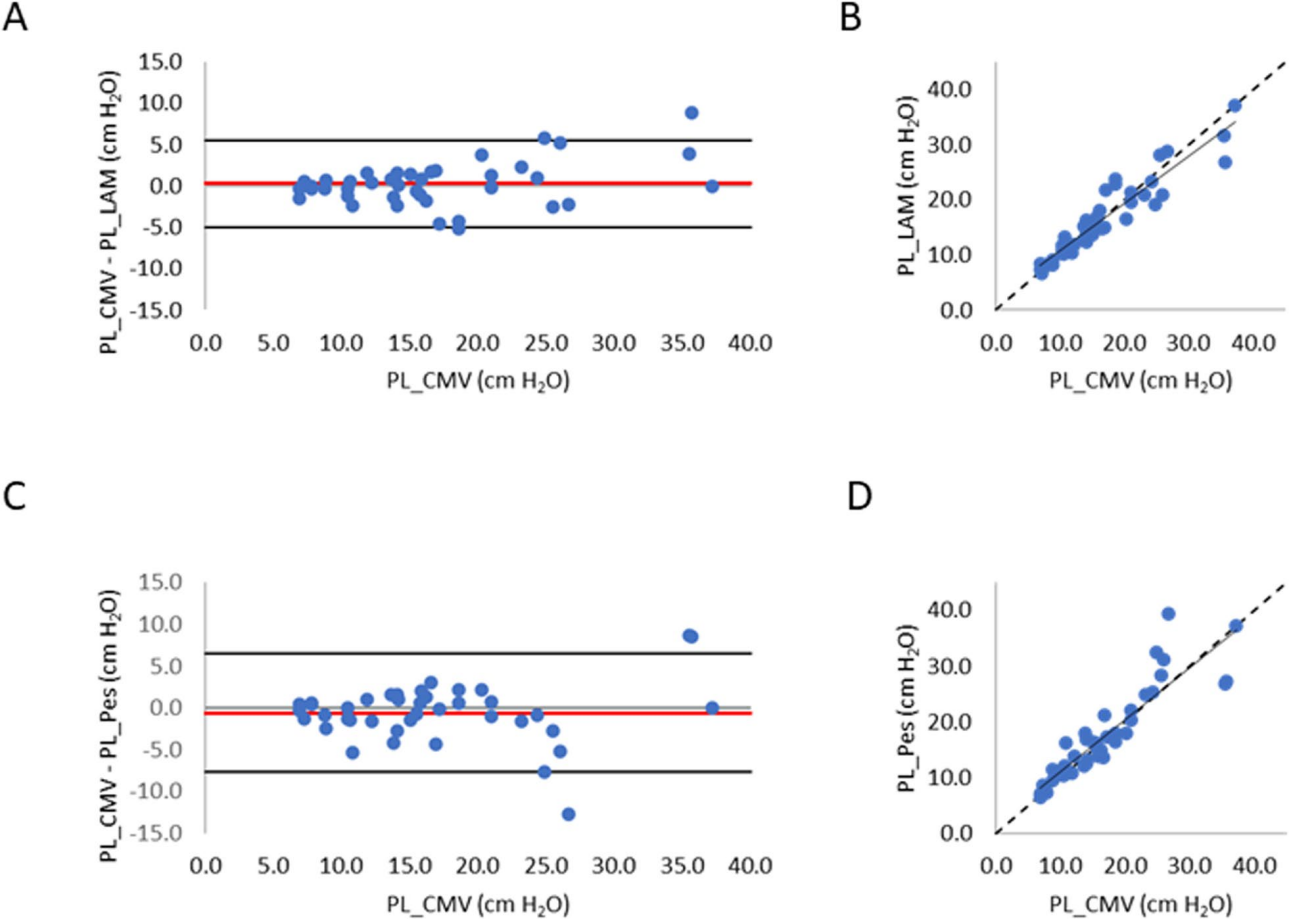
Bland–Altman plots and regression analysis for the group (*n* = 40 comparisons) comparing PL_CMV to PL_LAM and PL_Pes. **A** Difference between PL_LAM and PL_CMV (*y* axis) versus PL_CMV (*x* axis). Bias is indicated by the red solid line, and 1.96SD by the black solid horizontal lines. Same line colours for **C**. **B** Regression analysis between PL_LAM (*y* axis) and PL_CMV (*x* axis). Regression line (solid black line) and line of identity (black dashed line) are presented, also for **D**. **C** Difference between PL_Pes and PL_CMV (*y* axis) versus PL_CMV (*x* axis). **D** Regression analysis between PL_Pes (*y* axis) and PL_CMV (*x* axis)

As shown in Fig. 5, both PL_LAM (Panels A and B) and PL_Pes (Panels C and D) agreed well with PL_CMV. Presented are the 40 comparisons of the 15-min mean values obtained for two levels of assist during NAVA and NPS_SIM_ in the 10 patients. PL_LAM (y axis) was closely similar to PL_CMV (*x* axis), with a high correlation (*R*^2^ = 0.88) (Fig. 4B), *Y* = 0.86*x* + 2.10 (cmH_2_O). Bland–Altman analysis revealed a low bias of 0.28 cmH_2_O, and 1.96SD of 5.26 cmH_2_O (Fig. 5A). When all data were included in the Bland–Altman (for all 176 LAM breaths, Fig. E1A, B), the bias was reduced to 0.16 cmH_2_O, but with a larger dispersion (1.96SD was 7.54 cmH_2_O).

Also shown in Fig. 5, there was a good correlation between PL_Pes and PL_CMV (*R*^2^ = 0.81) Panel D, *Y* = 0.94*x* + 1.62 (cmH_2_O). Bland–Altman analysis revealed a bias of − 0.58 cmH_2_O (higher than PL_LAM) and a 1.96SD of 7.04 cmH_2_O (also higher than PL_LAM), Panel C. Figure E1D presents the data for all 176 LAM breaths and shows the same *R*^2^ = 0.81 (Panel D), with bias of 0.75 cmH_2_O and a 1.96SD of 7.92 cmH_2_O.

**Fig. 6:**
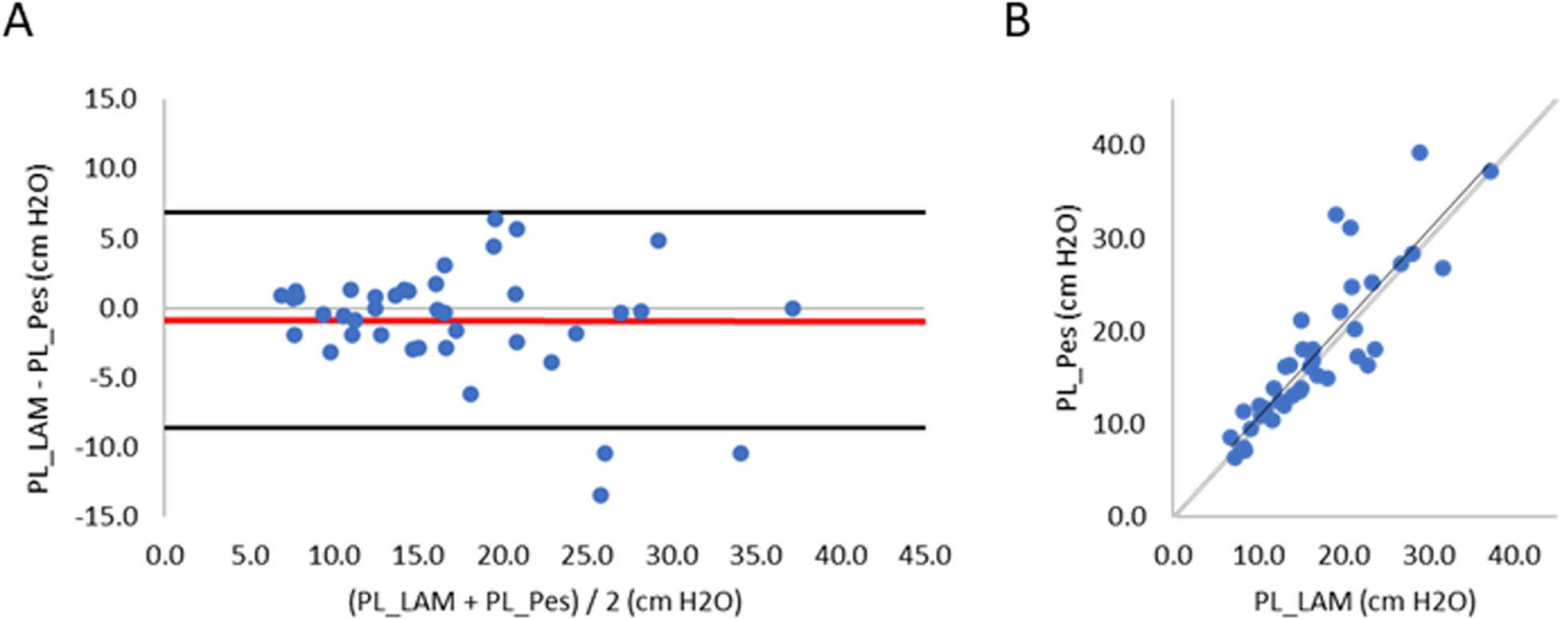
Bland–Altman plot and regression analysis for the group (*n* = 40 comparisons) comparing PL_LAM and PL_Pes. **A** Difference between PL_LAM and PL_Pes (*y* axis) versus the average of PL_LAM and PL_Pes (*x* axis). Bias is indicated by the red solid line, and 1.96SD by the black solid horizontal lines. **B** Regression analysis between PL_LAM (*x* axis) and PL_Pes (*y* axis). Regression line (solid black line) and line of identity (black dashed line) are presented

As shown in Fig. 6, PL_LAM and PL_Pes were similar to each other. Panel A reveals a low Bias (Bias = − 0.9 cmH_2_O) and 1.96SD of 7.71 cmH_2_O. Correlation between PL_LAM and PL_Pes was strong (*R*^2^ = 0.77).

For the matched breaths (assisted vs. controlled ventilation), tidal volume during CMV vs. tidal volume during NAVA/NPS_SIM_ correlated nearly perfectly (*R*^2^ = 0.97) and flow correlated well (*R*^2^ = 0.74).

Regression analysis showed that manual and automated analysis of PL_LAM were nearly identical for the 176 LAMS (*R*^2^ = 0.95) (Fig. E2A) as well as the averaged data (*n* = 40), *R*^2^ = 0.97 (Fig. E2B). This was the same finding for manual vs. automated analysis of PL_Pes: *R*^2^ = 0.97 and 0.99 for the 176 LAMS and averaged data (*n* = 40), respectively, Fig. E2C, D.

In one patient, PL_CMV was replaced by the average of PL_LAM and PL_Pes for the first three ventilation periods due to a change in airway resistance.

## Discussion

### Summary of the findings

This is the first physiological study to demonstrate that PL_LAM is a good estimate of transpulmonary driving pressure in patients who are spontaneously breathing on synchronized mechanical ventilation. There was a close correlation between PL_LAM and PL_CMV, as well as PL_LAM and PL_Pes, with a bias near zero, and low variability. In this study, we reproduced our previous findings demonstrated in sedated animals [2].

### Use of CMV and passive ventilation to confirm PL-estimates

Direct measurement of PL under conditions of passive ventilation was used as one of the “gold standards” in the present study. In each patient, we provided sedation and NMB to be able to ventilate without respiratory muscle involvement (confirmed by no Edi present). VC and PC settings were increased in a step-wise fashion, in small steps to provide a range of many flows and volumes that could later (in off-line analysis) be matched to the flows and volumes during NAVA and NPS_SIM_ at each assist level. Our assumption was that during paralyzed conditions, the pressure required to distend the lung to a given volume follows the equation of motion. Therefore, the biggest advantage of our study is that we “reproduced” the breathing pattern in CMV to match the flow and volume during assisted breaths. Bellani et al*.* conducted a study aiming at comparing the tidal change in transpulmonary pressure during assisted breathing and controlled ventilation, in a group of patients undergoing different levels of pressure support ventilation [3]. In their analysis, they found a good PL measurement only when matching breaths of similar volumes (*R*^2^ = 0.93), but they were challenged at matching flow (their flow correlation was weak *R*^2^ = 0.23). This is probably because their mode of choice for CMV was volume control (constant flow), whereas the assisted mode was PSV (descending flow). In our study, we realized from the previous animal work [2] that for “best matching” of assisted and CMV breaths, VC would be the best mode for matching NAVA (constant flow profile), and NPS_SIM_, PC breaths matched better (descending flow profile).

### Mathematical limitation of PL_LAM

A limitation of PL_LAM is that if the numerator (PIVent) is too small, or if the denominator approaches zero (when PVBC is high), PL_LAM estimates may reach mathematical uncertainty (Note, this does not necessarily imply clinical uncertainty). In the present study, we put a minimal limit of PIVent (numerator) which must be greater than 5 cmH_2_O for all PVBCs up to = 0.85. If PIVENT was 1–5 cmH_2_O, we allowed PVBC up to 0.90. These are not unreasonable criteria, as any patient on a pressure assist level less than 5 cmH_2_O implies minimal assist, and a large patient contribution to tidal volume. In fact, a PVBC > 0.90 basically approaches a situation where the patient is breathing on their own and contributing the same volume during the assisted breath as during the LAM. In the case of low PIVent and high PVBC, it could be useful to monitor the Edi at the same time to determine if the patient has a high respiratory drive due to under assist, or on the other hand if the Edi is within reasonable limits, could indicate readiness for extubation (patients are tolerating the low level of assist).

As previously discussed [2], similar neural drive (Edi) was required for the LAM and the assisted breath, to ensure that both breath types had similar diaphragm activation. Therefore, an Edi catheter was required to be in place for the current study. The LAM, from one breath to the next, is possible to obtain without much change to the diaphragm activation (it would take 3–5 extra breaths for Edi to respond [12]. Our inclusion criteria for “matched Edi” (slope, *r* value, intercept) are well in-line with previous recommendations [13].

### Limitations of the study

Similar to PL_LAM, PL_Pes also showed a close relationship to PL_CMV. One fundamental difference between PL_LAM and PL_Pes is the start and end time points used for waveform analysis. For PL_LAM comparisons to PL_CMV, the PL_CMV peak values were taken at the same volume where the spontaneous breaths had their peak Edi. For PL_Pes waveforms, the peak value could occur at anytime between the onset and end of the assisted breath (the timing of peak PL_Pes depends on respiratory mechanics and ventilator modes, flow profiles, and assist levels). Because the PL_Pes waveform can “turn around” and decline prior to peak Edi, if we had taken the measurement at peak Edi, PL_Pes could have been underestimated. The above arguments may also explain any (small) differences between PL_LAM and PL_Pes.

One artifact affecting Pes and therefore PL_Pes calculations is that the expiratory muscles can be recruited during the expiratory phase [14]. The subsequent relaxation of the contracted expiratory muscles (at the beginning of inspiration) can cause a negative deflection in Pes, unrelated to active inspiratory effort. In the present study, we limited this influence by applying criteria that excluded breaths where the baseline Pes differed between assisted and LAM breaths.

Only pre-inspiratory efforts resulting in negative airway pressure were considered to have a lung distending effect. Their deltas would be added to both PL_LAM and PL_Pes. As a result, this affected PL_Pes and PL_LAM’S relationship to PL_CMV equally and resulted in a close to zero bias.

A standard critique about manual analysis is personal “bias” affecting the results. The automated analysis and near-perfect correlation of manual vs. automated analysis for both PL_LAM and PL_Pes, minimized subjective influences (Fig. E2).

The presence of cardiac oscillations in the esophageal pressure [15] was not compensated for in the present study.

### Clinical implications

The present study was a physiological and feasibility evaluation of a maneuver (LAM) and a method for estimating transpulmonary driving pressure (PL_LAM) in patients on assisted ventilation. Therefore, clinical implications can only be suggestive until further studies and larger trials are published. The authors are confident however, that by combining PL_LAM, with knowledge about respiratory drive (Edi), ventilator pressure, flow and volume, there is a new possibility to investigate and monitor important phenomena as over-assist, ventilator-induced lung injury and patient self-inflicted lung injury.

## Conclusion

The findings of this study confirm our previous pre-clinical data and show that PL_LAM could offer a new tool for continuous monitoring of PL in spontaneously breathing patients on assisted mechanical ventilation.

## Supplementary Information

Supplementary Material 1.

## Data Availability

The datasets used and/or analyzed during the current study are available from the corresponding author on reasonable request.

## Abbreviations

LAM: Lower assist maneuver
PL: Transpulmonary driving pressure
NAVA: Neurally Adjusted Ventilatory Assist
NPS_SIM_: Neural Pressure Support (simulated)
CMV: Controlled mechanical ventilation
PL_CMV: Transpulmonary driving pressure during controlled mechanical ventilation
PL_LAM: Transpulmonary driving pressure estimated with LAM
PL_Pes: Transpulmonary driving pressure calculated with esophageal pressure
PVent: Ventilator-delivered pressure
Pes: Esophageal pressure
Edi: Diaphragm electrical activity
Pga: Gastric pressure
PVBC: Patient-ventilator breath contribution
PIVent: Inflation pressure of ventilator, calculated as difference in PVent (assist) − PVent (LAM)
VC: Volume control mode
PC: Pressure control mode
PEEP: Positive end-expiratory pressure
Paw: Airway pressure
