# Supplementary material for: Estimation of transpulmonary driving pressure using a lower assist maneuver (LAM) during synchronized ventilation in patients with acute respiratory failure: a physiological study
Source: Intensive Care Med Exp. 2024 Oct 4;12:89. doi: 10.1186/s40635-024-00674-z (PMC11452363; doi:10.1186/s40635-024-00674-z)
Supplement: Supplementary file 1 — Supplementary Material 1. [file 40635_2024_674_MOESM1_ESM.docx]

**ELECTRONIC SUPPLEMENTARY MATERIAL**

**Estimation of Transpulmonary Driving Pressure Using a Lower Assist Maneuver (LAM) during Synchronized Ventilation in Patients with Acute Respiratory Failure: A Physiological Study**

Ling Liu, MD PhD^1^; Hao He MD^1^; Meihao Liang MD^1^; *Jennifer Beck PhD^2,3,4^; Christer Sinderby PhD^2,4,5^

^1^Jiangsu Provincial Key Laboratory of Critical Care Medicine, Department of Critical Care Medicine, Zhongda Hospital, School of Medicine, Southeast University, Nanjing, 210009, China

^2^Keenan Research Centre for Biomedical Science of St. Michael’s Hospital; Department of Critical Care, St. Michael's Hospital, Toronto, Canada

^3^Department of Pediatrics, University of Toronto, Toronto, Canada

^4^Member, Institute for Biomedical Engineering and Science Technology (iBEST) at Ryerson University and St-Michael’s Hospital, Toronto, Canada

^5^Department of Medicine and Interdepartmental Division of Critical Care Medicine, University of Toronto, Toronto, Canada

**Table of Contents**

**Page 1.** Title page

**Page 2.** TOC

**Page 3.** Methods

**Page 4.** Results

**Pages 5-6.** Figures E1-E2 with legends

**Methods**

Steps for calculation of respiratory system compliance and resistance for Table 1

In each patient, and for each ventilator period:

1. Flow and volume from the assisted breaths were determined
2. VC breaths (measured under NMB) with corresponding flow and volume from (i) were selected for calculation of respiratory system (static) compliance and resistance.
3. From the VC breath that had corresponding flow and volume, we determined the peak ventilator pressure and the plateau pressure.
4. Compliance = Volume/(plateau pressure -PEEP).
5. Resistance = (peak pressure-plateau pressure)/flow

**Results**

The average “Edi matching” regression results between LAM breaths and assisted breaths for the automated analysis were R^2^ = 0.93 ± 0.01, slope = 0.96 ± 0.04, and intercept -0.05 ± 0.30 mV.

Regarding manual analysis of “matching” CMV breaths to assisted breaths, the average number of matched breaths per 15-min ventilation period was 41.9 breaths (SD 7.6; range 24-53 breaths).

**Figure E1** demonstrates Bland-Altman plots and regression analysis for all LAM breaths that had comparisons (n = 176) between PL_CMV and PL_LAM (**Panels A and B**), and PL_CMV and PL_Pes (**Panels C and D**).

Compared to Figure 5 (n=40) of the main manuscript, when all data was included (**Figure E1, Panel A**), the comparison of PL_LAM and PL_CMV showed that the bias was 0.16 cm H_2_O with limits of agreement of 1.96 x the standard deviation (1.96SD) of 7.54 cm H_2_O.

Regarding PL_Pes, **Figure E1, Panels C and D** also present the data for all 176 LAM breaths and shows R^2^ of 0.81 (**Panel D**) with bias of 0.75 cm H_2_O and a 1.96SD of 7.92 cm H_2_O (**Panel C**).

Comparing manual and automated analyses showed that PL_LAM values were nearly identical for the 176 LAMS (R^2^ = 0.95) (**Figure E2, Panel A**) as well as for the 40 periods of averaged data (R^2^ = 0.97) (**Figure E2, Panel B**). The same findings were observed for manual vs. automated analysis of PL_Pes: R^2^ = 0.97 and 0.99 for the 176 LAMS and averaged data, respectively, **Figure E2, Panels C and D**.

**FIGURE E1**

**
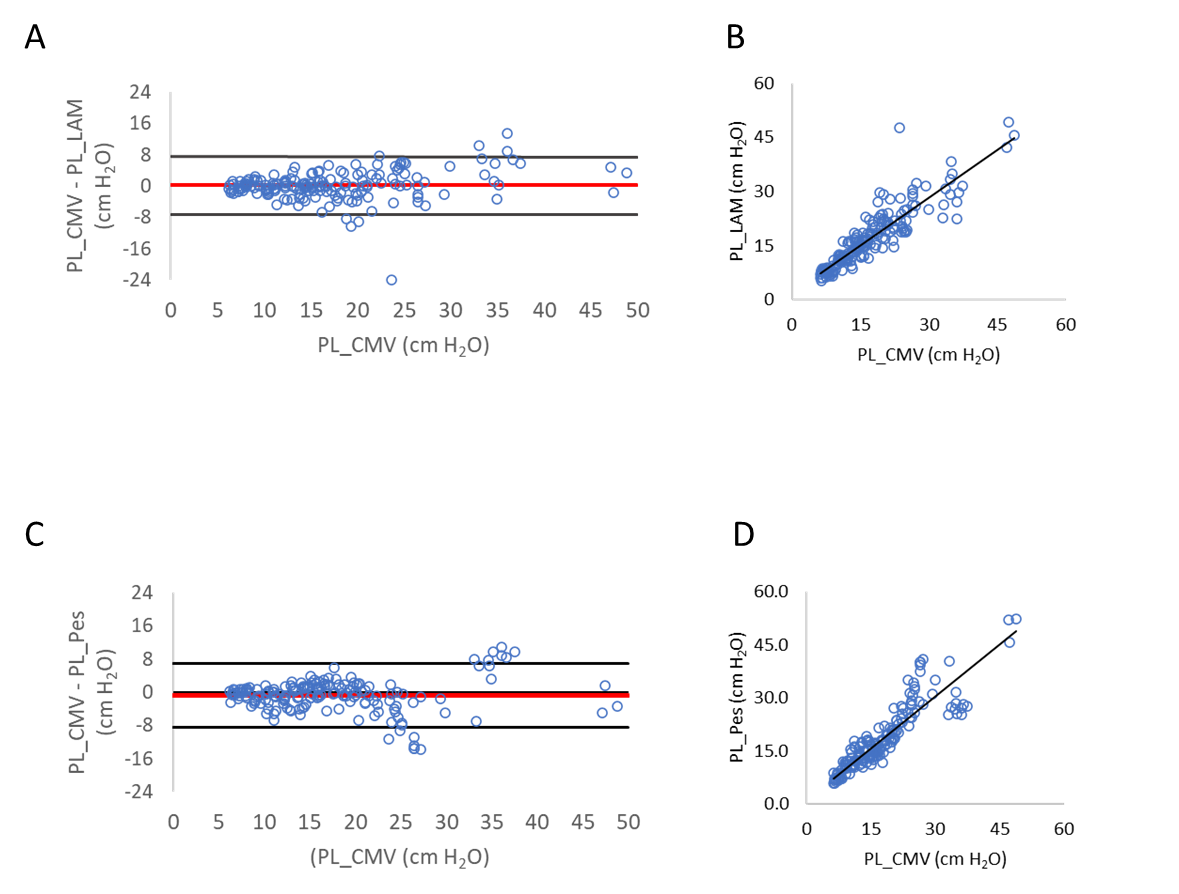
**

**FIGURE E1: Bland-Altman plots and regression analysis for all comparisons (all LAM breaths that had comparisons, n = 176, 3 min averages) between PL_CMV and PL_LAM, and PL_CMV and PL_Pes**.

Panel A: Difference between PL_LAM and PL_CMV (y axis) versus PL_CMV (x axis). Red lines indicate the bias, and horizontal solid black lines indicate 1.96SD. Panel B: Regression analysis between PL_LAM (y axis) and PL_CMV (x axis). Panel C: Difference between PL_Pes and PL_CMV (y axis) versus PL_CMV (x axis). Panel D: Regression analysis between PL_Pes (y axis) and PL_CMV (x axis).

**FIGURE E2**

**
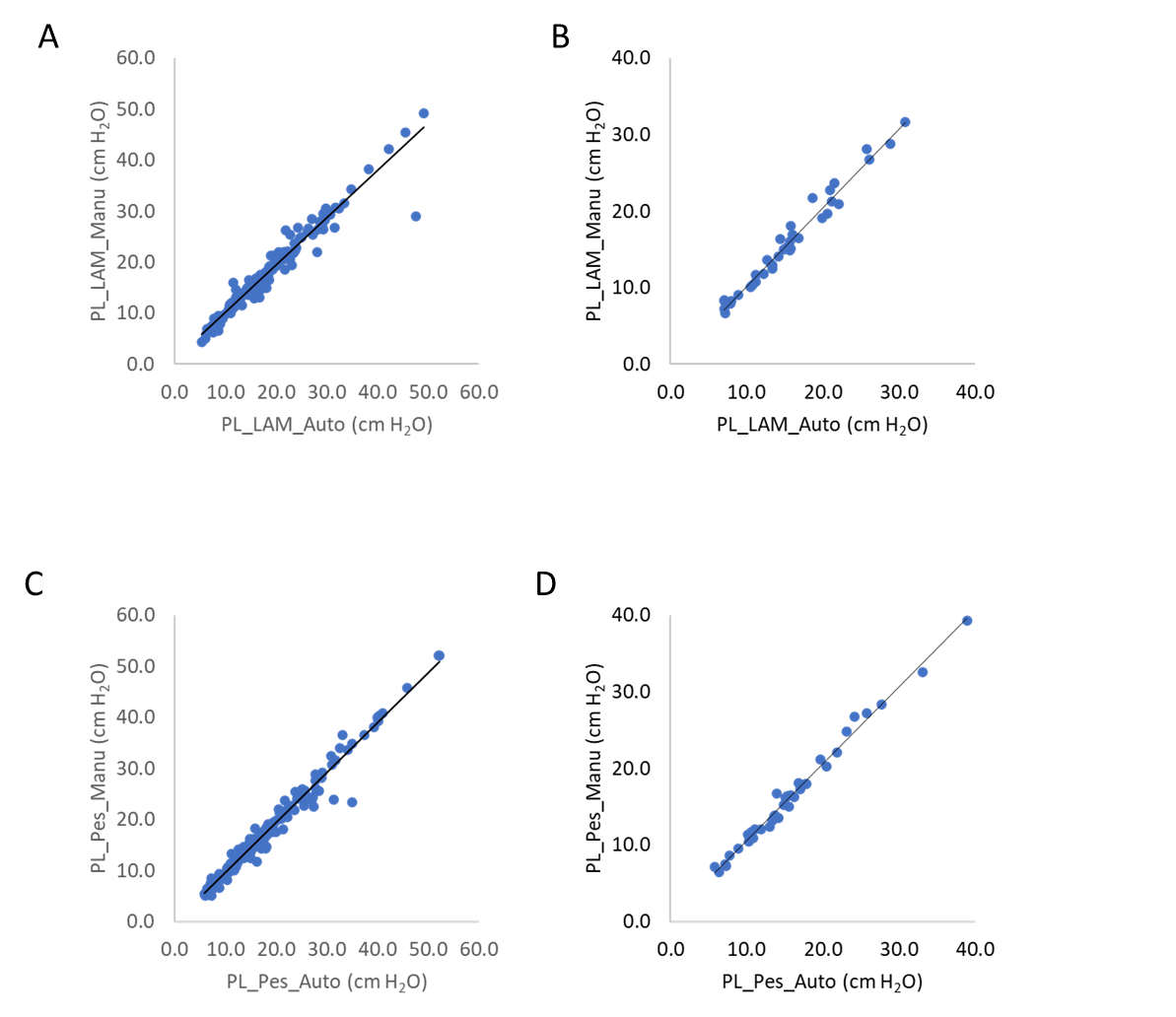
**

**FIGURE E2: Comparison of Manual versus Automated analysis**

Panel A: Relationship between manual analysis (y axis) and automated analysis (x axis) for PL_LAM (Panels A and B) and PL_Pes (Panels C and D). Left panels show all individual LAM maneuvers (n=176) analyzed and right panels show averaged values of all LAM maneuvers at each level of assist with PSV and NAVA (n=40). Note that all relationships are near identical suggesting that manual analysis was not affected by analyst.
